# ‘Firm’ foundations: Restoring apprenticeship‐style learning for today's students

**DOI:** 10.1111/medu.15661

**Published:** 2025-03-05

**Authors:** Holly Harper, Thomas Agar, Stefanie Berkes, Reeza Khan, Quentin Mak

## 1 WHAT PROBLEMS WERE ADDRESSED?

To become a doctor is to join a professional community. Yet, this community feeling can be lost in large medical programmes. At our medical school, the largest in the UK, final‐year students expressed how disconnection within and between year groups negatively impacts their sense of belonging and professional growth. The ‘firm’ system^1^ once provided this hidden curriculum through team integration and mentorship. However, the shift away from this apprentice‐style learning model means students lack consistent guidance for identity development in the clinical setting. With upcoming expansion of medical school places, we advocate the value of student‐led role modelling in professional identity formation

## 2 WHAT WAS TRIED?

We created the ‘2to5 Peer Teaching Programme’ to address this issue. A total of 52 final‐year students facilitated weekly small‐group teaching, across four teaching hospitals, for 134 second‐year students in their first clinical year.

The ‘2to5’ was incorporated into the formal teaching schedule, endorsed by faculty. Facilitators developed their own resources, hosting lectures, bedside teaching, practical sessions and tutorial‐style discussions. When teaching was out‐of‐hours, students were motivated to attend as learning objectives were tailored to their requests.

Experienced lead volunteers with pre‐existing familiarity with their hospital sites supervised sessions and implemented site‐specific organisation, overcoming administrative challenges by liaising with administrative teams in‐person and identifying informal teaching spaces when necessary.

## 3 WHAT LESSONS WERE LEARNED?

The ‘2to5’ demonstrates the value of senior medical students assuming a sustained mentorship role, beyond traditional near‐peer teaching.

Students described it as ‘the best teaching I've got’, highlighting ‘older students…have a better perspective on our situation’ and ‘you can trust that this is stuff…we need to know’. Student‐reported preparedness for the future, on Likert scales, increased from an average of 4.59 to 8.28 (*n* = 93), where 1 was feeling not prepared at all and 10 was feeling extremely prepared.

Thematic analysis of focus groups was undertaken through coding via inductive data reduction. This uncovered a theme of inspiration and empowerment, with students describing an improved sense of belonging and confidence in the clinical environment. They particularly valued guidance on how to maximise placement opportunities, such as consenting patients for clinical examinations and knowing who to approach on new wards. They described a greater sense of optimism for the future, stating: ‘It's nice to…know that everyone's in a similar position at this stage and you get more confident as you progress’. Students reported the customisable nature compensated for differences in teaching provision between large, tertiary placements and smaller sites. Interestingly, students did not feel facilitator continuity influenced their programme satisfaction, likely because they had a close relationship with the entire team. This aligns with the ‘firm’ structure, where continuity is maintained through a group rather than individuals.

Areas for improvement included implementation earlier in the year to enable earlier professional role‐modelling and an easier transition into placement. Encouraging facilitators to share their research, quality improvement and teaching experiences would meet student desire for formal career mentorship.

The ‘2to5’ reconceptualises the ‘firm’ model mentorship aspect, which is challenged by increasing medical student populations. We champion this as a logistically sustainable educational intervention for professional identity formation in Medicine.

All authors contributed to the initial drafting and revision of the manuscript and agreed to be accountable for all aspects of the work.

## AUTHOR CONTRIBUTIONS

**Holly Harper:** Conceptualization; writing – original draft; project administration; data curation. **Thomas Agar:** Data curation; formal analysis; project administration; writing – original draft. **Stefanie Berkes:** Conceptualization; project administration; writing – original draft; investigation. **Reeza Khan:** Conceptualization; project administration; writing – original draft; investigation. **Quentin Mak:** Writing – review and editing; project administration.

## CONFLICT OF INTEREST STATEMENT

No conflict of interest declared.

## ETHICS STATEMENT

This work was carried out in accordance with the Declaration of Helsinki. Informed consent was obtained for all participants of the focus groups and surveys, with data anonymity guaranteed. Ethical considerations were made by Dr Alice Roueché, Deputy Head of Stage, who supervised the project.

## Data Availability

The data that support the findings of this study are available from the corresponding author upon reasonable request.
